# CHILDHOOD FAMILY ENVIRONMENT, GENETIC RISK FOR ALZHEIMER’S DISEASE, AND LATE-LIFE COGNITIVE DECLINE

**DOI:** 10.1093/geroni/igad104.2131

**Published:** 2023-12-21

**Authors:** Shiyang Zhang, Sae Hwang Han

**Affiliations:** The University of Texas at Austin, Austin, Texas, United States; University of Texas at Austin, Austin, Texas, United States

## Abstract

How genetic risks for Alzheimer’s disease (AD) interact with environmental factors to shape individuals’ cognitive trajectories across the lifespan is a burgeoning area of research. However, relatively little is known about the role childhood family environment plays in the relationship between genetic risk factors for AD and cognitive decline in later life. This study examined whether and how early-life family environments may modify cognitive decline associated with genetic AD risks. Using longitudinal data from the Health and Retirement Study (HRS; 1998-2018), we followed over 9,000 individuals aged 51 and older during the observation period spanning nearly two decades. Cognitive functioning was assessed with the full cognitive battery from the HRS. Childhood family environment was evaluated separately with the relationship quality with one’s mother and the number of household adults before the age of 16. We estimated a series of multilevel models to assess the association between polygenic risk score for AD (PGS-AD) and changes in cognitive functioning, and whether the association was moderated by the two measures of childhood social capital. Findings indicated that childhood maternal relationship quality, but not the number of household adults, protected against cognitive decline accelerated by PGS-AD. That is, the associations between higher PGS-AD and accelerated cognitive decline were attenuated for individuals who reported a better childhood maternal relationship. Findings are in line with the biopsychosocial model of health and suggest that a better quality of maternal relationships in childhood may have a protective effect on cognitive functioning across the lifespan.

